# The Type 2 Diabetes Associated Minor Allele of rs2237895 *KCNQ1* Associates with Reduced Insulin Release Following an Oral Glucose Load

**DOI:** 10.1371/journal.pone.0005872

**Published:** 2009-06-11

**Authors:** Johan Holmkvist, Karina Banasik, Gitte Andersen, Hiroyuki Unoki, Thomas Skot Jensen, Charlotta Pisinger, Knut Borch-Johnsen, Annelli Sandbæk, Torsten Lauritzen, Sören Brunak, Shiro Maeda, Torben Hansen, Oluf Pedersen

**Affiliations:** 1 Hagedorn Research Institute, Gentofte, Denmark; 2 Laboratory for Endocrinology and Metabolism, Center for Genomic Medicine, RIKEN, Yokohama, Kanagawa, Japan; 3 Center for Biological Sequence Analysis, Technical University of Denmark, Lyngby, Denmark; 4 Research Centre for Prevention and Health, Glostrup University Hospital, Glostrup, Denmark; 5 Steno Diabetes Center, Gentofte, Denmark; 6 Faculty of Health Science, University of Aarhus, Aarhus, Denmark; 7 Department of General Practice, University of Aarhus, Aarhus, Denmark; 8 Faculty of Health Science, University of Southern Denmark, Odense, Denmark; 9 Faculty of Health Science, University of Copenhagen, Copenhagen, Denmark; University of Bremen, Germany

## Abstract

**Background:**

Polymorphisms in the potassium channel, voltage-gated, KQT-like subfamily, member 1 (*KCNQ1*) have recently been reported to associate with type 2 diabetes. The primary aim of the present study was to investigate the putative impact of these *KCNQ1* polymorphisms (rs2283228, rs2237892, rs2237895, and rs2237897) on estimates of glucose stimulated insulin release.

**Methodology/Principal Findings:**

Genotypes were examined for associations with serum insulin levels following an oral glucose tolerance test (OGTT) in a population-based sample of 6,039 middle-aged and treatment-naïve individuals. Insulin release indices estimated from the OGTT and the interplay between insulin sensitivity and insulin release were investigated using linear regression and Hotelling T2 analyses.

Applying an additive genetic model the minor C-allele of rs2237895 was associated with reduced serum insulin levels 30 min (mean±SD: (CC) 277±160 vs. (AC) 280±164 vs. (AA) 299±200 pmol/l, p = 0.008) after an oral glucose load, insulinogenic index (29.6±17.4 vs. 30.2±18.7vs. 32.2±22.1, p = 0.007), incremental area under the insulin curve (20,477±12,491 vs. 20,503±12,386 vs. 21,810±14,685, p = 0.02) among the 4,568 individuals who were glucose tolerant. Adjustment for the degree of insulin sensitivity had no effect on the measures of reduced insulin release. The rs2237895 genotype had a similar impact in the total sample of treatment-naïve individuals. No association with measures of insulin release were identified for the less common diabetes risk alleles of rs2237892, rs2237897, or rs2283228.

**Conclusion:**

The minor C-allele of rs2237895 of *KCNQ1*, which has a prevalence of about 42% among Caucasians was associated with reduced measures of insulin release following an oral glucose load suggesting that the increased risk of type 2 diabetes, previously reported for this variant, likely is mediated through an impaired beta cell function.

## Introduction

Type 2 diabetes is a common complex disorder, characterised by chronic hyperglycemia as the result of an incapacity of the pancreatic beta cells to compensate for the degree of insulin resistance [1]. Glucose-stimulated insulin secretion is biphasic; impaired or absent first-phase insulin secretion is an early feature of type 2 diabetes, while second-phase insulin secretion deteriorates during progression of the disease. Biphasic insulin secretion is triggered by electrical signalling in the beta cell as a result of a functional interplay between K_ATP_ channels, K_V_-channels and voltage-dependent Ca^2+^ channels [2]–[7]. Hence, genes involved in maintaining and regulating the electrogradient in the beta cells are plausible candidate genes for type 2 diabetes. To date, genetic variation in voltage-dependent Ca^2+^ channels (*CACNA1E*) and K_ATP_ (*KCNJ11*) channels have been shown to influence insulin secretion and type 2 diabetes risk [8], [9], and genome-wide association studies (GWAS) for type 2 diabetes in Caucasians have proven the importance of genes coding for proteins involved in insulin secretion [10]–[14]. Two independent GWAS in Japanese individuals have identified a novel type 2 diabetes gene: the potassium channel, voltage-gated, KQT-like subfamily, member 1 (*KCNQ1*), and for the two studies, the association was replicated in Danish and Singaporean individuals (meta-analysis: rs2237895 OR = 1.23 (1.18–1.29), p<1.0×10^−16^ and rs2237897 OR = 1.33 (1.24–1.41), p<1.0×10^−16^) [15], and in Chinese, Korean and Swedish individuals (meta-analysis: rs2237895 OR = 1.31 (1.25–138), p = 6.1×10^−26^, and rs2237892 OR = 1.40 (1.34–1.47), p<1.7×10^−42^ [16].

*KCNQ1* is located on chromosome 11p15.5, a region that also contains other genes which have previously been associated with type 2 diabetes, e.g., *CDKN1C* [17]. Linkage to type 2 diabetes has similarly been identified at chromosome 11p12–p13 in a Japanese study [18]. Mutations in *KCNQ1* are known to cause the autosomal-recessive and -dominant forms of the long QT-syndrome (Jervell and Lange-Nielsen [19] and Romano-Ward [20]), and common variation has also been genome-wide associated with altered QT interval [21], [22]. *KCNQ1* encodes the pore-forming α-subunit of the I_Ks_-channel (K_V_7.1) which is expressed in the human heart and pancreas as well as in the kidney, placenta, liver, lung, and intestine [15], [23]. The basal pore of the K_V_7.1-channel consists of four KCNQ1 subunits, which assemble with different KCNE β-subunit family members (e.g., KCNE1 in cardiac tissue and KCNE3 in colonic tissue) to form protein complexes with different potassium current properties [24]. From studies in INS-1 cells it has been suggested that KCNQ1 assembles with KCNE2 in insulin-secreting cells, and that blocking of the KCNQ1 K^+^ channel with the sulphonamide analogue 293B reduces whole beta cell outward currents with 60%, and that the insulin secretion significantly increases in the presence of both 293B and tolbutamide [25].

To test the hypothesis that the recently reported type 2 diabetes-associated variants in *KCNQ1* have an effect on insulin release, we investigated rs2283228, rs2237892, rs2237895, and rs2237897 for association with serum insulin levels during an oral glucose tolerance test (OGTT) in a population-based sample of 6,039 middle-aged and treatment-naïve Danes.

## Results

### Effect of the common KCNQ1 rs2237895 on measures of serum insulin release

Four *KCNQ1* polymorphisms (rs2237892, rs2283228, rs2237895, and rs2237897) previously shown to associate with type 2 diabetes [15], [16] were genotyped in 6,164 Danes (Table S1), who were part of the Danish case-control sample in the study by Unoki *et al.* [15]. These variants were investigated for an association with type 2 diabetes-related quantitative traits in the population-based Inter99 study sample involving 6,039 treatment-naïve middle-aged individuals of whom 4,568 were normal glucose tolerant according to WHO criteria. Three of the variants (rs2237892, rs2283228, and rs2237897) were not associated with type 2 diabetes related quantitative traits (Tables S2–[Supplementary-material pone.0005872.s003] [Supplementary-material pone.0005872.s004] [Supplementary-material pone.0005872.s005] [Supplementary-material pone.0005872.s006] S7). However, both glucose tolerant individuals and treatment-naïve study participants of Inter99 with the minor C-allele of *KCNQ1* rs2237895 (minor allele frequency = 42.5%) had significantly lower measures of serum insulin and serum C-peptide release under an additive genetic model (Table 1, 2). No differences in insulin resistance (HOMA-IR) were observed (Table 1, 2). Data on D′ and r^2^ for the four SNPs are given in (Table S8).

**Table 1:** Anthropometrics and quantitative metabolic traits in 4,239 successfully genotyped individuals with normal glucose tolerance from the population-based Inter99 study sample in relation to the rs2237895 genotypes of *KCNQ1*.

**rs2237895**				
	AA	AC	CC	P_additive_
N (m/w)	1,489 (689/800)	2,073 (972/1101)	677 (308/369)	
Age (years)	45±8	45±8	46±8	
BMI (kg/m^2^)	25.6±4.0	25.5±4.1	25.5±4.2	0.45
HOMA-IR	9.0±5.8	8.9±5.7	8.7±5.3	0.25
**Glucose traits**				
Fasting p-glucose (mmol/l)	5.3±0.4	5.3±0.4	5.3±0.4	0.31
p-glucose at 30 min (mmol/l)	8.2±1.6	8.2±1.5	8.2±1.5	0.78
p-glucose at 120 min (mmol/l)	5.5±1.1	5.5±1.1	5.5±1.1	0.79
incAUC glucose	181±103	180±99	185±101	0.62
**Insulin traits**				
Fasting s-insulin (pmol/l)	38±24	37±24	37±22	0.31
s-insulin at 30 min (pmol/l)	299±200	280±164	277±160	0.0076
s-insulin at 120 min (pmol/l)	172±136	165±126	166±135	0.38
incAUC insulin	21,810±14,685	20,503±12,386	20,477±12,491	0.015
Fasting s-C-peptide (pmol/l)	537±201	540±224	535±208	0.89
C-peptide at 30 min (pmol/l)	2,014±704	1,957±681	1,950±702	0.022
C-peptide at 120 min (pmol/l)	2,064±788	2,051±795	2,048±811	0.42
incAUC C-peptide (pmol/l)	157,265±53,026	153,022±51,967	152,965±53,118	0.045
Insulinogenic index	32±22	30±19	30±17	0.0065
Disposition index	4.3±3.1	4.1±2.9	4.1±2.7	0.069
BIGTT-S_I_	10±4	10±4	11±4	0.051
BIGTT-AIR	1,950±1,179	1,875±964	1,846±951	0.04

The table includes unadjusted mean±S.D data. P-values shown are for an additive genetic model and are adjusted for age, BMI and sex. incAUC, incremental area under the curve; HOMA-IR, homeostasis model assessment of insulin resistance; BIGTT-SI, BIGTT-insulin sensitivity; BIGTT-AIR, BIGTT acute insulin response.

**Table 2:** Anthropometrics and quantitative metabolic traits of a total of 5,597 successfully genotyped middle-aged and treatment-naïve individuals from the Inter99 sample including the 4,239 glucose-tolerant individuals presented in Table 1.

**rs2237895**					
	AA	AC	CC	P_additive_	
N (m/w)	1,933 (940/993)	2,721 (1,373/1,348)	943 (480/463)		
Age (years)	46±8	46±8	47±8		
BMI (kg/m^2^)	26.2±4.5	26.2±4.5	26.4±4.8	0.66	
HOMA-IR	10.5±7.6	10.5±8.1	10.9±8.5	0.75	
**Glucose traits**					
Fasting p-glucose (mmol/l)	5.5±0.8	5.5±0.7	5.6±0.9	0.068	
p-glucose at 30 min (mmol/l)	8.7±1.9	8.7±1.8	8.9±2.0	0.30	
p-glucose at 120 min (mmol/l)	6.1±2.0	6.2±2.1	6.3±2.2	0.18	
incAUCglucose	219±134	220±135	228±136	0.46	
**Insulin traits**					
Fasting s-insulin (pmol/l)	42±27	42±28	42±28	0.50	
s-insulin at 30 min (pmol/l)	302±198	286±179	284±169	0.0011	
s-insulin at 120 min (pmol/l)	219±217	215±208	218±217	0.68	
incAUC insulin	23,743±16,727	22,500±15,554	22,604±15,572	0.0061	
Fasting s-C-peptide (pmol/l)	588±255	597±283	607±286	0.46	
C-peptide at 30 min (pmol/l)	2,032±713	1,984±722	1,983±716	0.013	
C-peptide at 120 min (pmol/l)	2,304±1,003	2,307±1,030	2,320±1,012	0.84	
incAUC C-peptide	163,740±57,671	160,006±57,933	159,661±58,165	0.035	
Insulinogenic index	30.7±21.3	28.8±19.0	28.1±17.5	0.00073	
Disposition index	3.8±3.0	3.6±2.8	3.5±2.6	0.0097	
BIGTT-S_I_	9.2±4.0	9.3±4.1	9.1±4.2	0.55	
BIGTT-AIR	1,892±1,140	1,841±1,062	1,784±948	0.0083	

Study participants are stratified according to rs2237895 genotypes of *KCNQ1*.

P-values shown are for an additive genetic model and are adjusted for age, BMI and sex. incAUC, incremental area under the curve; HOMA-IR, homeostasis model assessment of insulin resistance; BIGTT-SI, BIGTT-insulin sensitivity; BIGTT-AIR, BIGTT acute insulin response.

**Figure 1:**
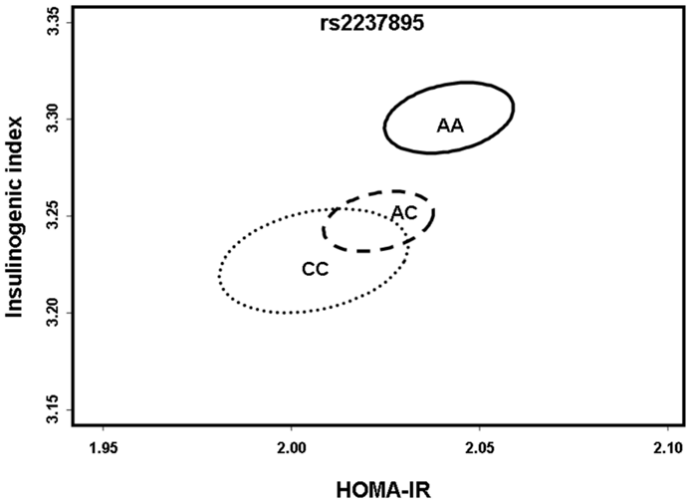
Multivariate analysis on the effect of the minor C-allele of *KCNQ1* rs2237895 on insulin release in response to the level of insulin sensitivity in 4,568 glucose tolerant individuals from Inter99. The multivariate method, Hotelling's T^2^ [36], was applied to test the simultaneous effect of genotype on insulinogenic index and HOMA-IR for rs2237895. Two-dimensional standard error of the means of each genotype level for insulinogenic index and HOMA-IR were calculated for *KCNQ1* rs2237895. Significant multivariate association with the minor C-allele of rs2237895 was detected under an additive genetic model (p = 0.004) suggesting that the association with insulin release was not dependent on the level of insulin sensitivity but a true beta cell abnormality.

In order to further investigate a putative beta cell abnormality, the interplay between insulin release (Insulinogenic index: I/G30), insulin resistance (Homeostasis model assessment of insulin resistance: HOMA-IR) and the genetic predisposition to type 2 diabetes with *KCNQ1* rs2237895, we applied the multivariate Hotelling's T^2^ method to simultaneously test the effect of genotype on I/G30 and HOMA-IR in the sample of glucose tolerant individuals. Significant multivariate association with the rs2237895 C-risk-allele and the combination of I/G30 and HOMA-IR was demonstrated (p = 0.004; p_dominant_ = 0.004) (Figure 1).

## Discussion

Current knowledge of the KCNQ1 protein in INS-1-cells and the association of the *KCNQ1* polymorphisms with type 2 diabetes in two large Japanese studies [15], [16] led us to investigate a role for these variants in type 2 diabetes-related quantitative traits (especially serum insulin release) in the population-based Inter99 study sample. Individuals with the minor C-allele of *KCNQ1* rs2237895 had significantly reduced estimates of first-phase insulin release as measured by serum insulin concentration at 30 min and I/G30 and the association was not dependent on the level of insulin sensitivity suggesting a true beta cell abnormality (Figure 1).

The K_V_-channels are believed to play an important role in the pancreatic beta cells mediating repolarisation of the membrane terminating Ca^2+^-influx and insulin secretion, and a K_V_-channel knock-out in rat islets as well as pharmacological inhibition of K_V_-channels in mouse beta cells have been reported to enhance glucose-stimulated insulin secretion [4]–[7]. The K_V_7.1 channel, encoded by *KCNQ1*, is expressed in INS-1 cells and has been suggested to play an important role in maintaining the membrane potential in these cells [25]. Based on *in vitro* data and the association with type 2 diabetes, there is compelling evidence suggesting an effect also on type 2 diabetes-related quantitative traits with variation in this gene. In context of the known function of the protein encoded by *KCNQ1* it is also interesting to note the potential relationship between an increased risk of sudden cardiac death with unknown etiology in individuals with diabetes [26], supported by recent studies showing genome-wide significant association with common variation in *KCNQ1* and QT-interval of the ECG [21], [22], as well as association with the Mendelian long QT syndrome and sudden death [27].

**Figure 2:**
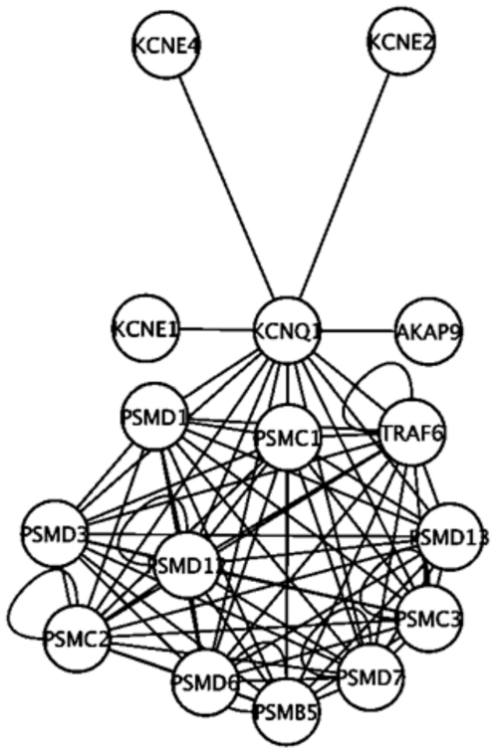
KCNQ1 -protein interaction analyses as estimated from bioinformatics-driven protein-protein network analyses including interactions transferred from other organisms by orthology. PSMC x - Proteasome 26S subunit, ATPase, x; PSMD x – Proteasome 26S subunit, ATPase, x; PSMB5 – Proteasome subunit, beta-type, 5; AKAP9 – A-Kinase Anchor Protein 9; KCNE x – Potassium Channel, Voltage-gated, ISK-related Subfamily, Member x.

Furthermore, an in silico protein-protein interaction network analyses, performed as in [28], suggested several interesting protein interactions and pathways that could potentially affect insulin secretion, e.g., *AKAP9*, encoding the Yotiao-protein (Figure 2). This protein has previously been reported to form a large macromolecular complex with KCNQ1 important in coordinating cAMP-dependent PKA phosphorylation of the KCNQ1-channel [29]. A mutation in *AKAP9* has also been shown to cause long QT syndrome subtype 11 by disrupting the binding to KCNQ1 leading to reduced cAMP-stimulated PKA phosphorylation of the KCNQ1-channel and a prolonged repolarization period [30]. In addition, anchoring of PKA to AKAPs is involved in GLP-1-mediated but not glucose-mediated insulin secretion [31].

In recent studies [15], [16] large differences in allele frequencies were observed for *KCNQ1* rs2237892, rs2283228, and rs2237897 between Japanese and Scandinavian individuals. Given the low allele frequency for these three variants in our Caucasian population the statistical power to identify an association with type 2 diabetes related quantitative traits with an equal effect size as in the Japanese studies was low. This fact might explain the lack of association with measures of serum insulin release for these variants. However, it could also indicate that these variants are not causative but rather good proxies for the causative variant/s in the Japanese population and poorer proxies in individuals of Scandinavian ancestry. This given, we cannot exclude that rs2237895 is in strong LD with a causative variant/s in this or in another nearby gene in this heavily imprinted and gene-dense region since the selection of these variants were based on previous findings linking them to an increased risk of type 2 diabetes. In this context it is important to notice that polymorphisms in the neighbouring *CDKN1C* have been associated with increased birth weight [17], and that variation in the *CDKN* family (*CDKN2A/B* locus; chromosome 9p21) has been associated with type 2 diabetes in recent GWAS- and GWAS-replication studies [10]–[12], [14], [32]. Thus, given the large allele-frequency differences of *KCNQ1* variants observed in previous studies [15], [16], genotyping and re-sequencing of the *KCNQ1* locus and nearby loci on chromosome 11p in additional populations may help identifying the causative variant/s explaining the regional association with reduced insulin release and type 2 diabetes. Using the overly conservative Bonferroni correction the present data would not hold for multiple testing, but since the measures of insulin release during the OGTT are highly correlated we believe such corrections are too conservative. Although Tan *et al*. [33] have also reported association of variation in the *KCNQ1* locus and impaired beta-cell function in 3734 Asians additional replication attempts in large independent studies with careful measurements of stimulated insulin release are warranted.

In conclusion, we report insulin sensitivity independent impairment of insulin release following an oral glucose load in a large population of middle-aged treatment naïve individuals carrying the type 2 diabetes associated minor C-allele of *KCNQ1* rs2237895.

## Materials and Methods

### Subjects

The four polymorphisms (rs2283228, rs2237892, rs2237895, rs2237897) were genotyped in 6,164 Danes from the population-based Inter99 study sample [34] (Table S1). The glucose tolerance status of these participants were characterised according to WHO criteria [1]; normal glucose tolerance (n = 4,568), impaired fasting glycaemia (n = 508), impaired glucose tolerance (n = 707), or screen-detected T2D (n = 256); 125 had known treated type 2 diabetes and were excluded from the quantitative trait analyses. The glucose tolerant individuals from the Inter99 study sample were part of the Danish control group in the previously published Japanese *KCNQ1* case-control study [15].

### Ethics statement

All participants were of Danish nationality and informed written consent was obtained from all participants before participation. The studies were approved by the Ethical Committee of Copenhagen and were in accordance with the principle of the Helsinki Declaration II.

### Biochemical and anthropometric measurements

Height and body weight were measured in light indoor clothes and without shoes, and BMI was calculated as weight (kg) divided by height squared (m^2^). Waist circumference was measured in the standing position midway between the iliac crest and the lower costal margin and hip circumference at its maximum. In the Inter99 participants blood samples were drawn after a 12-h overnight fast. Plasma glucose was analysed by a glucose oxidase method (Granutest, Merck, Darmstadt, Germany), HbA_1C_ was measured by ion-exchange high-performance liquid chromatography (normal reference range: 0.041–0.064) and serum insulin (excluding des(31, 32) and intact proinsulin) was measured using the AutoDELFIA insulin kit (Perkin-Elmer/Wallac, Turku, Finland). Serum C-peptide concentrations were measured by a time-resolved fluoroimmunoassay (AutoDELFIA C-peptide kit; Perkin-Elmer/Wallac, Turku, Finland). BIGTT-insulin sensitivity index (BIGTT-S_I_) and BIGTT-acute insulin response (BIGTT-AIR) use information on sex and BMI combined with analysis of plasma glucose and serum insulin levels at time points 0, 30, and 120 min during an OGTT to provide indices for S_I_ and AIR that highly correlate with these indices obtained during an intra-venous glucose tolerance test. These indices were calculated as described elsewhere [35]. Insulinogenic index (I/G30) is an index of first phase insulin release during an oral glucose challenge and was calculated as fasting serum insulin subtracted from serum insulin at 30 min [pmol/l] divided by plasma glucose at 30 min [mmol/l]. Insulin resistance was determined by the Homeostasis model assessment of insulin resistance (HOMA-IR) and calculated as fasting plasma glucose [mmol/l] multiplied by fasting serum insulin [pmol/l] and divided by 22.5. The disposition index is an index of insulin release in response to insulin resistance and was calculated as I/G30 divided by HOMA-IR. The area under curve for plasma glucose, serum insulin and serum C-peptide were calculated using the trapezoidal method.

### Bioinformatics-driven protein-protein interaction analyses

In an attempt to detect proteins that interact with KCNQ1 we applied protein-protein interaction analyses, performed as detailed in [28].

### Genotyping

Genotyping was performed using Taqman allelic discrimination (KBiosciences, Herts, UK) with a success rate >96%. Discordance was <0.4% as judged from re-genotyping of 966 random duplicate samples. Allele frequencies were in accordance with HapMap (CEU) data (rs2237895 has not been genotyped in the CEU population) and obeyed Hardy-Weinberg equilibrium (p>0.4).

### Statistical analyses

A general linear model was used to test for an association with quantitative variables in the groups of normal glucose tolerant and treatment-naïve individuals with abnormal glucose regulation (impaired fasting glucose, impaired glucose tolerance and screen-detected type 2 diabetes). Non-normally distributed data (measures of serum insulin release and C-peptides, HOMA-IR, insulinogenic index, disposition index and BIGTT) were logarithmically transformed before analyses. All analyses were adjusted for age, BMI, and sex.

The multivariate method, Hotelling T^2^ [36], was applied to test the simultaneous effect of genotype on serum insulin release (I/G30) and insulin sensitivity (HOMA-IR) in the Inter99 sample of non-diabetic individuals.

The statistical power to detect a difference in serum insulin at 30 min was estimated using simulations. We assumed an additive genetic model for both the simulation of the data and for the testing of the data using a linear model. We used the empirical variance of the observed traits to simulate phenotypes from a normal distribution so that the variance across genotypes is drawn from the estimated variance. The power was estimated using 5,000 simulations and with a significance threshold of α = 0.05. Based on the different allele frequencies and the 4,568 normal glucose-tolerant individuals, we estimated the effect sizes per allele of quantitative traits for which we had 80 and 90% statistical power, respectively, to detect an association. Depending on allele frequency (4.0–42.5%) and assuming an additive model, we had 80% statistical power to detect an allele-dependent difference of 7.4–2.9% for serum insulin 30 min and 8.1–3.2% for I/G30. Similarly, we had 90% statistical power to detect an 8.4–3.4% and 9.4–3.7% change per allele in serum insulin 30 min and I/G30, respectively.

The statistical analyses were performed using R version 2.7.2 (available at http://www.r-project.org), SPSS (version 14.0, Chicago, IL, USA) and PLINK [37]. P-values were not adjusted for multiple hypothesis testing and a p-value of <0.05 was considered statistically significant.

## Supporting Information

Table S1 Clinical characteristics of study participants. Data are means±standard deviation. NGT, normal glucose tolerance, IFG, impaired fasting glucose, IGT, impaired glucose tolerance, T2D, type 2 diabetes.
(0.02 MB DOC)

Table S2 Anthropometrics and quantitative metabolic traits among normal-glucose tolerant participants in the population-based Inter99 study sample in relation to the rs2237897 genotypes of *KCNQ1*. The table includes unadjusted mean±S.D data for a total of 4,375 middle-aged individuals with normal glucose tolerance stratified according to genotype. P-values shown are for an additive genetic model and are adjusted for age, BMI and sex. incAUC, incremental area under the curve; HOMA-IR, homeostasis model assessment of insulin resistance; BIGTT-SI, BIGTT-insulin sensitivity; BIGTT-AIR, BIGTT acute insulin response.
(0.03 MB DOC)

Table S3 Anthropometrics and quantitative metabolic traits among normal-glucose tolerant participants in the population-based Inter99 study sample in relation to the rs2283228 genotypes of *KCNQ1*. The table includes unadjusted mean±S.D data for a total of 4,381 middle-aged individuals with normal glucose tolerance stratified according to genotype. P-values shown are for an additive genetic model and are adjusted for age, BMI and sex. incAUC, incremental area under the curve; HOMA-IR, homeostasis model assessment of insulin resistance; BIGTT-SI, BIGTT-insulin sensitivity; BIGTT-AIR, BIGTT acute insulin response.
(0.03 MB DOC)

Table S4 Anthropometrics and quantitative metabolic traits among normal-glucose tolerant participants in the population-based Inter99 study sample in relation to the rs2237892 genotypes of *KCNQ1*. The table includes unadjusted mean±S.D data for a total of 4,381 middle-aged individuals with normal glucose tolerance stratified according to genotype. P-values shown are for an additive genetic model and are adjusted for age, BMI and sex. incAUC, incremental area under the curve; HOMA-IR, homeostasis model assessment of insulin resistance; BIGTT-SI, BIGTT-insulin sensitivity; BIGTT-AIR, BIGTT acute insulin response.
(0.03 MB DOC)

Table S5 Anthropometrics and quantitative metabolic traits in the population-based Inter99 study sample in relation to the rs2237897 genotypes of *KCNQ1*. Data are unadjusted mean±S.D data for a total of 5,776 middle-aged individuals with either normal glucose tolerance (n = 4,375), impaired fasting glycemia (n = 485), impaired glucose tolerance (n = 667) or screen-detected and treatment-naïve type 2 diabetes (n = 249) stratified according to genotype. General linear regression analyses were used to calculate differences between geneotypes and p-values shown are for an additive genetic model and are adjusted for age, BMI and sex. incAUC, incremental area under the curve; HOMA-IR, homeostasis model assessment of insulin resistance; BIGTT-SI, BIGTT-insulin sensitivity; BIGTT-AIR, BIGTT acute insulin response.
(0.03 MB DOC)

Table S6 Anthropometrics and quantitative metabolic traits in the population-based Inter99 study sample in relation to the rs2283228 genotypes of *KCNQ1*. Data are unadjusted mean±S.D data for a total of 5,787middle-aged individuals with either normal glucose tolerance (n = 4,381), impaired fasting glycemia (n = 491), impaired glucose tolerance (n = 667) or screen-detected and treatment-naïve type 2 diabetes (n = 248) stratified according to genotype. General linear regression analyses were used to calculate differences between geneotypes and p-values shown are for an additive genetic model and are adjusted for age, BMI and sex. incAUC, incremental area under the curve; HOMA-IR, homeostasis model assessment of insulin resistance; BIGTT-SI, BIGTT-insulin sensitivity; BIGTT-AIR, BIGTT acute insulin response.
(0.03 MB DOC)

Table S7 Anthropometrics and quantitative metabolic traits in the population-based Inter99 study sample in relation to the rs2237892 genotypes of *KCNQ1*. Data are unadjusted mean±S.D data for a total of 5,781 middle-aged individuals with either normal glucose tolerance (n = 4,381), impaired fasting glycemia (n = 483), impaired glucose tolerance (n = 672) or screen-detected and treatment-naïve type 2 diabetes (n = 245) stratified according to genotype. General linear regression analyses were used to calculate differences between geneotypes and p-values shown are for an additive genetic model and are adjusted for age, BMI and sex. incAUC, incremental area under the curve; HOMA-IR, homeostasis model assessment of insulin resistance; BIGTT-SI, BIGTT-insulin sensitivity; BIGTT-AIR, BIGTT acute insulin response.
(0.03 MB DOC)

Table S8 D′ and r2 measures for the investigated *KCNQ1* SNPs. Top triangle gives D′ and bottom triangle gives r2-values.
(0.02 MB DOC)
