# Supplementary material for: The Type 2 Diabetes Associated Minor Allele of rs2237895 KCNQ1 Associates with Reduced Insulin Release Following an Oral Glucose Load
Source: PLoS One. 2009 Jun 11;4(6):e5872. doi: 10.1371/journal.pone.0005872 (PMC2689931; doi:10.1371/journal.pone.0005872)
Supplement: Table S1 — Clinical characteristics of study participants. Data are means±standard deviation. NGT, normal glucose tolerance, IFG, impaired fasting glucose, IGT, impaired glucose tolerance, T2D, type 2 diabetes. (0.02 MB DOC) [file pone.0005872.s001.doc]

**Table S1: Clinical characteristics of study participants.**

|  | Inter99 | | |
| --- | --- | --- | --- |
|  | NGT | IFG and IGT | Type 2 diabetes patients |
| N (m/w) | 4,568 (2122/2446) | 1,215 (721/494) | 381 (227/154) |
| Age (years) | 45.2 ± 7.8 | 48.7 ± 7.3 | 50.8 ± 7.2 |
| BMI (kg/m^2^) | 25.5 ± 4.1 | 28.0 ± 4.9 | 30.0 ± 5.7 |
| HbA1_c_ (%) | 5.8 ± 0.4 | 5.9 ± 0.4 | 7.1 ± 1.7 |

Data are means ± standard deviation. NGT, normal glucose tolerance, IFG, impaired fasting glucose, IGT, impaired glucose tolerance, T2D, type 2 diabetes.
