# Supplementary material for: The Type 2 Diabetes Associated Minor Allele of rs2237895 KCNQ1 Associates with Reduced Insulin Release Following an Oral Glucose Load
Source: PLoS One. 2009 Jun 11;4(6):e5872. doi: 10.1371/journal.pone.0005872 (PMC2689931; doi:10.1371/journal.pone.0005872)
Supplement: Table S2 — Anthropometrics and quantitative metabolic traits among normal-glucose tolerant participants in the population-based Inter99 study sample in relation to the rs2237897 genotypes of KCNQ1. The table includes unadjusted mean±S.D data for a total of 4,375 middle-aged individuals with normal glucose tolerance stratified according to genotype. P-values shown are for an additive genetic model and are adjusted for age, BMI and sex. incAUC, incremental area under the curve; HOMA-IR, homeostasis model assessment of insulin resistance; BIGTT-SI, BIGTT-insulin sensitivity; BIGTT-AIR, BIGTT acute insulin response. (0.03 MB DOC) [file pone.0005872.s002.doc]

Table S2: Anthropometrics and quantitative metabolic traits among normal-glucose tolerant participants in the population-based Inter99 study sample in relation to the rs2237897 genotypes of *KCNQ1*.

| **rs2237897** | | | | |
| --- | --- | --- | --- | --- |
|  | CC | CT | TT | P _additive_ |
| N (m/w) | 3,999 (1,860/2,139) | 367 (161/206) | 9 (4/5) |  |
| Age (years) | 45±8 | 45±8 | 42±7 |  |
| BMI (kg/m^2^) | 25.5±4.1 | 25.4±4.1 | 24.8±2.6 | 0.77 |
| HOMA-IR | 8.9±5.7 | 9.1±5.5 | 6.6±4.6 | 0.25 |
| **Glucose traits** | | | | |
| Fasting p-glucose (mmol/l) | 5.3±0.4 | 5.3±0.4 | 4.8±0.7 | 0.028 |
| p-glucose at 30 min (mmol/l) | 8.2±1.5 | 8.1±1.6 | 7.7±1.8 | 0.51 |
| p-glucose at 120 min (mmol/l) | 5.5±1.1 | 5.4±1.1 | 4.6±1.0 | 0.015 |
| incAUC glucose | 182±101 | 176±99 | 159±133 | 0.36 |
| **Insulin traits** | | | | |
| Fasting s-insulin (pmol/l) | 37±23 | 38±23 | 31±20 | 0.13 |
| s-insulin at 30 min (pmol/l) | 285±175 | 296±179 | 338±222 | 0.065 |
| s-insulin at 120 min (pmol/l) | 168±132 | 167±113 | 123±69 | 0.94 |
| incAUC insulin | 20,876±13,229 | 21,480±12,483 | 21,420±11,132 | 0.19 |
| Fasting s-C-peptide (pmol/l) | 540±215 | 534±184 | 466±130 | 1.0 |
| C-peptide at 30 min (pmol/l) | 1,974±693 | 2,007±682 | 2,012±739 | 0.27 |
| C-peptide at 120 min (pmol/l) | 2,060±800 | 2,037±750 | 1,781±434 | 0.57 |
| incAUC C-peptide (pmol/l) | 154,485±52,627 | 155,928±51,510 | 152,083±40,166 | 0.59 |
| Insulinogenic index | 31±19 | 32±21 | 40±27 | 0.045 |
| Disposition index | 4.2±2.9 | 4.2±2.7 | 6.7±3.5 | 0.70 |
| BIGTT-S_I_ | 10±4 | 10±4 | 13±3 | 0.73 |
| BIGTT-AIR | 1,887±1,021 | 1,947±1,110 | 2,450±983 | 0.16 |

The table includes unadjusted mean±S.D data for a total of 4,375 middle-aged individuals with normal glucose tolerance stratified according to genotype. P-values shown are for an additive genetic model and are adjusted for age, BMI and sex. incAUC, incremental area under the curve; HOMA-IR, homeostasis model assessment of insulin resistance; BIGTT-SI, BIGTT-insulin sensitivity; BIGTT-AIR, BIGTT acute insulin response.
