# Supplementary material for: The Type 2 Diabetes Associated Minor Allele of rs2237895 KCNQ1 Associates with Reduced Insulin Release Following an Oral Glucose Load
Source: PLoS One. 2009 Jun 11;4(6):e5872. doi: 10.1371/journal.pone.0005872 (PMC2689931; doi:10.1371/journal.pone.0005872)
Supplement: Table S3 — Anthropometrics and quantitative metabolic traits among normal-glucose tolerant participants in the population-based Inter99 study sample in relation to the rs2283228 genotypes of KCNQ1. The table includes unadjusted mean±S.D data for a total of 4,381 middle-aged individuals with normal glucose tolerance stratified according to genotype. P-values shown are for an additive genetic model and are adjusted for age, BMI and sex. incAUC, incremental area under the curve; HOMA-IR, homeostasis model assessment of insulin resistance; BIGTT-SI, BIGTT-insulin sensitivity; BIGTT-AIR, BIGTT acute insulin response. (0.03 MB DOC) [file pone.0005872.s003.doc]

Table S3: Anthropometrics and quantitative metabolic traits among normal-glucose tolerant participants in the population-based Inter99 study sample in relation to the rs2283228 genotypes of *KCNQ1*.

| **rs2283228** | | | | |
| --- | --- | --- | --- | --- |
|  | AA | AC | CC | P _additive_ |
| N (m/w) | 3,719 (1,715/2,004) | 633 (294/339) | 29 (18/11) |  |
| Age (years) | 45±8 | 45±8 | 42±8 |  |
| BMI (kg/m^2^) | 25.5±4.1 | 25.5±4.1 | 25.5±4.1 | 0.86 |
| HOMA-IR | 8.9±5.7 | 9.0±5.3 | 9.5±7.5 | 0.27 |
| **Glucose traits** | | | | |
| Fasting p-glucose (mmol/l) | 5.3±0.4 | 5.3±0.4 | 5.1±0.6 | 0.27 |
| p-glucose at 30 min (mmol/l) | 8.2±1.5 | 8.2±1.7 | 8.0±1.2 | 0.63 |
| p-glucose at 120 min (mmol/l) | 5.5±1.1 | 5.5±1.1 | 4.9±1.2 | 0.075 |
| incAUC glucose | 180±101 | 183±105 | 165±87 | 0.84 |
| **Insulin traits** | | | | |
| Fasting s-insulin (pmol/l) | 37±23 | 38±22 | 41±32 | 0.19 |
| s-insulin at 30 min (pmol/l) | 284±175 | 289±175 | 366±210 | 0.071 |
| s-insulin at 120 min (pmol/l) | 168±132 | 167±121 | 145±81 | 0.85 |
| incAUC insulin | 20,857±13,260 | 21,045±12833 | 24,545±11,911 | 0.18 |
| Fasting s-C-peptide (pmol/l) | 539±215 | 540±198 | 551±193 | 0.36 |
| C-peptide at 30 min (pmol/l) | 1,971±695 | 1,991±653 | 2,250±747 | 0.073 |
| C-peptide at 120 min (pmol/l) | 2,058±800 | 2,052±766 | 1,897±605 | 0.93 |
| incAUC C-peptide (pmol/l) | 154,320±52,782 | 154,890±50,164 | 162,919±42,803 | 0.33 |
| Insulinogenic index | 31±19 | 31±20 | 40±23 | 0.094 |
| Disposition index | 4.2±2.9 | 4.1±2.9 | 5.5±3.6 | 0.70 |
| BIGTT-S_I_ | 10±4 | 10±4 | 10±4 | 0.64 |
| BIGTT-AIR | 1,885±1,005 | 1,923±1,212 | 2,432±1,099 | 0.20 |

The table includes unadjusted mean±S.D data for a total of 4,381 middle-aged individuals with normal glucose tolerance stratified according to genotype. P-values shown are for an additive genetic model and are adjusted for age, BMI and sex. incAUC, incremental area under the curve; HOMA-IR, homeostasis model assessment of insulin resistance; BIGTT-SI, BIGTT-insulin sensitivity; BIGTT-AIR, BIGTT acute insulin response.
