# Supplementary material for: The Type 2 Diabetes Associated Minor Allele of rs2237895 KCNQ1 Associates with Reduced Insulin Release Following an Oral Glucose Load
Source: PLoS One. 2009 Jun 11;4(6):e5872. doi: 10.1371/journal.pone.0005872 (PMC2689931; doi:10.1371/journal.pone.0005872)
Supplement: Table S4 — Anthropometrics and quantitative metabolic traits among normal-glucose tolerant participants in the population-based Inter99 study sample in relation to the rs2237892 genotypes of KCNQ1. The table includes unadjusted mean±S.D data for a total of 4,381 middle-aged individuals with normal glucose tolerance stratified according to genotype. P-values shown are for an additive genetic model and are adjusted for age, BMI and sex. incAUC, incremental area under the curve; HOMA-IR, homeostasis model assessment of insulin resistance; BIGTT-SI, BIGTT-insulin sensitivity; BIGTT-AIR, BIGTT acute insulin response. (0.03 MB DOC) [file pone.0005872.s004.doc]

Table S4: Anthropometrics and quantitative metabolic traits among normal-glucose tolerant participants in the population-based Inter99 study sample in relation to the rs2237892 genotypes of *KCNQ1*.

| **rs2237892** | | | | |
| --- | --- | --- | --- | --- |
|  | CC | CT | TT | P _additive_ |
| N (m/w) | 3,833 (1,770/2,063) | 530 (249/281) | 18 (9/9) |  |
| Age (years) | 45±8 | 45±8 | 39±7 |  |
| BMI (kg/m^2^) | 25.5±4.1 | 25.5±4.0 | 24.4±3.1 | 0.72 |
| HOMA-IR | 8.9±5.7 | 9.0±5.5 | 8.1±5.3 | 0.40 |
| **Glucose traits** | | | | |
| Fasting p-glucose (mmol/l) | 5.3±0.4 | 5.3±0.4 | 5.0±0.7 | 0.32 |
| p-glucose at 30 min (mmol/l) | 8.2±1.5 | 8.3±1.6 | 7.9±1.4 | 0.47 |
| p-glucose at 120 min (mmol/l) | 5.5±1.1 | 5.4±1.1 | 5.0±1.3 | 0.053 |
| incAUC glucose | 181±101 | 182±103 | 174±97 | 0.78 |
| **Insulin traits** | | | | |
| Fasting s-insulin (pmol/l) | 37±23 | 38±23 | 37±23 | 0.30 |
| s-insulin at 30 min (pmol/l) | 284±174 | 292±184 | 343±171 | 0.14 |
| s-insulin at 120 min (pmol/l) | 168±132 | 164±116 | 148±78 | 0.60 |
| incAUC insulin | 20,877±13,169 | 21,093±13,045 | 23,355±7,795 | 0.31 |
| Fasting s-C-peptide (pmol/l) | 540±215 | 539±197 | 495±176 | 0.80 |
| C-peptide at 30 min (pmol/l) | 1,973±691 | 1,998±688 | 2,136±659 | 0.22 |
| C-peptide at 120 min (pmol/l) | 2,060±799 | 2,045±770 | 1,825±573 | 0.63 |
| incAUC C-peptide (pmol/l) | 154,444±52,741 | 155,034±51,279 | 159,261±27,521 | 0.47 |
| Insulinogenic index | 31±19 | 32±21 | 39±20 | 0.17 |
| Disposition index | 4.2±2.9 | 4.1±2.8 | 5.9±3.8 | 0.78 |
| BIGTT-S_I_ | 10±4 | 10±4 | 11±3 | 0.97 |
| BIGTT-AIR | 1,885±1,011 | 1,929±1,183 | 2,377±913 | 0.34 |

The table includes unadjusted mean±S.D data for a total of 4,381 middle-aged individuals with normal glucose tolerance stratified according to genotype. P-values shown are for an additive genetic model and are adjusted for age, BMI and sex. incAUC, incremental area under the curve; HOMA-IR, homeostasis model assessment of insulin resistance; BIGTT-SI, BIGTT-insulin sensitivity; BIGTT-AIR, BIGTT acute insulin response.
