# Supplementary material for: The Type 2 Diabetes Associated Minor Allele of rs2237895 KCNQ1 Associates with Reduced Insulin Release Following an Oral Glucose Load
Source: PLoS One. 2009 Jun 11;4(6):e5872. doi: 10.1371/journal.pone.0005872 (PMC2689931; doi:10.1371/journal.pone.0005872)
Supplement: Table S5 — Anthropometrics and quantitative metabolic traits in the population-based Inter99 study sample in relation to the rs2237897 genotypes of KCNQ1. Data are unadjusted mean±S.D data for a total of 5,776 middle-aged individuals with either normal glucose tolerance (n = 4,375), impaired fasting glycemia (n = 485), impaired glucose tolerance (n = 667) or screen-detected and treatment-naïve type 2 diabetes (n = 249) stratified according to genotype. General linear regression analyses were used to calculate differences between geneotypes and p-values shown are for an additive genetic model and are adjusted for age, BMI and sex. incAUC, incremental area under the curve; HOMA-IR, homeostasis model assessment of insulin resistance; BIGTT-SI, BIGTT-insulin sensitivity; BIGTT-AIR, BIGTT acute insulin response. (0.03 MB DOC) [file pone.0005872.s005.doc]

Table S5: Anthropometrics and quantitative metabolic traits in the population-based Inter99 study sample in relation to the rs2237897 genotypes of *KCNQ1*.

| **rs2237897** | | | | |
| --- | --- | --- | --- | --- |
|  | CC | CT | TT | P _additive_ |
| N (m/w) | 5,290 (2,638/2,652) | 474 (228/246) | 12 (6/6) |  |
| Age (years) | 46±8 | 46±8 | 43±8 |  |
| BMI (kg/m^2^) | 26.2±4.5 | 26±4.4 | 25.7±4.0 | 0.46 |
| HOMA-IR | 10.6±8 | 10.7±8.7 | 10.7±13.8 | 0.38 |
| **Glucose traits** | | | | |
| Fasting p-glucose (mmol/l) | 5.5±0.8 | 5.5±0.7 | 5.0±0.8 | 0.021 |
| p-glucose at 30 min (mmol/l) | 8.7±1.9 | 8.6±1.9 | 7.9±1.9 | 0.38 |
| p-glucose at 120 min (mmol/l) | 6.2±2.1 | 6.1±2.0 | 5.6±2.0 | 0.048 |
| IncAUCglucose | 221±136 | 214±131 | 194±145 | 0.36 |
| Insulin traits | | | | |
| Fasting s-insulin (pmol/l) | 42±28 | 43±28 | 45±29 | 0.16 |
| s-insulin at 30 min (pmol/l) | 290±184 | 303±181 | 354±252 | 0.013 |
| s-insulin at 120 min (pmol/l) | 217±213 | 215±195 | 279±511 | 0.74 |
| IncAUC insulin | 22,861±16,039 | 23,317±14,461 | 29,852±32,109 | 0.098 |
| Fasting s-C-peptide (pmol/l) | 597±274 | 591±271 | 539±274 | 0.94 |
| C-peptide at 30 min (pmol/l) | 1,996±718 | 2,054±714 | 2,017±877 | 0.061 |
| C-peptide at 120 min (pmol/l) | 2,313±1,020 | 2,297±1,016 | 2,273±1,449 | 0.85 |
| IncAUC C-peptide | 161,035±57,967 | 163,885±58,339 | 162,188±77,105 | 0.29 |
| Insulinogenic index | 29.1±19.5 | 31.1±20.3 | 38.2±26.3 | 0.017 |
| Disposition index | 3.6±2.8 | 3.8±2.6 | 5.4±3.9 | 0.38 |
| BIGTT-S_I_ | 9.2±4.1 | 9.3±4 | 11.6±5.1 | 0.44 |
| BIGTT-AIR | 1,840±1,081 | 1,889±1,062 | 2,401±929 | 0.076 |

Data are unadjusted mean±S.D data for a total of 5,776 middle-aged individuals with either normal glucose tolerance (n = 4,375), impaired fasting glycemia (n = 485), impaired glucose tolerance (n = 667) or screen-detected and treatment-naïve type 2 diabetes (n = 249) stratified according to genotype. General linear regression analyses were used to calculate differences between geneotypes and p-values shown are for an additive genetic model and are adjusted for age, BMI and sex. incAUC, incremental area under the curve; HOMA-IR, homeostasis model assessment of insulin resistance; BIGTT-SI, BIGTT-insulin sensitivity; BIGTT-AIR, BIGTT acute insulin response.
