# Supplementary material for: The Type 2 Diabetes Associated Minor Allele of rs2237895 KCNQ1 Associates with Reduced Insulin Release Following an Oral Glucose Load
Source: PLoS One. 2009 Jun 11;4(6):e5872. doi: 10.1371/journal.pone.0005872 (PMC2689931; doi:10.1371/journal.pone.0005872)
Supplement: Table S6 — Anthropometrics and quantitative metabolic traits in the population-based Inter99 study sample in relation to the rs2283228 genotypes of KCNQ1. Data are unadjusted mean±S.D data for a total of 5,787middle-aged individuals with either normal glucose tolerance (n = 4,381), impaired fasting glycemia (n = 491), impaired glucose tolerance (n = 667) or screen-detected and treatment-naïve type 2 diabetes (n = 248) stratified according to genotype. General linear regression analyses were used to calculate differences between geneotypes and p-values shown are for an additive genetic model and are adjusted for age, BMI and sex. incAUC, incremental area under the curve; HOMA-IR, homeostasis model assessment of insulin resistance; BIGTT-SI, BIGTT-insulin sensitivity; BIGTT-AIR, BIGTT acute insulin response. (0.03 MB DOC) [file pone.0005872.s006.doc]

Table S6: Anthropometrics and quantitative metabolic traits in the population-based Inter99 study sample in relation to the rs2283228 genotypes of *KCNQ1*.

| **rs2283228** | | | | |
| --- | --- | --- | --- | --- |
|  | AA | AC | CC | P _additive_ |
| N (m/w) | 4,947 (2,455/2,492) | 806 (402/404) | 34 (20/14) |  |
| Age (years) | 46±8 | 46±8 | 43±9 |  |
| BMI (kg/m^2^) | 26.2±4.5 | 26.1±4.5 | 26±4.5 | 0.37 |
| **Glucose traits** | | | | |
| Fasting p-glucose (mmol/l) | 5.5±0.8 | 5.5±0.7 | 5.2±0.6 | 0.0077 |
| p-glucose at 30 min (mmol/l) | 8.7±1.9 | 8.7±1.9 | 8.2±1.3 | 0.49 |
| p-glucose at 120 min (mmol/l) | 6.2±2.1 | 6.1±2.1 | 5.5±1.8 | 0.017 |
| IncAUC glucose | 221±136 | 216±134 | 196±117 | 0.39 |
| **Insulin traits** | | | | |
| Fasting s-insulin (pmol/l) | 42±28 | 41±26 | 50±44 | 0.71 |
| s-insulin at 30 min (pmol/l) | 290±184 | 293±181 | 368±214 | 0.076 |
| s-insulin at 120 min (pmol/l) | 219±214 | 206±190 | 228±334 | 0.30 |
| IncAUC insulin | 22,935±16,085 | 22,460±14,637 | 27,996±20,570 | 0.52 |
| Fasting s-C-peptide (pmol/l) | 598±276 | 586±258 | 609±314 | 0.91 |
| C-peptide at 30 min (pmol/l) | 1,997±720 | 2,012±685 | 2,246±791 | 0.071 |
| C-peptide at 120 min (pmol/l) | 2,320±1,028 | 2,256±952 | 2,185±1,115 | 0.33 |
| IncAUC C-peptide (pmol/l) | 161,335±58,321 | 159,869±54,632 | 169,919±55,395 | 0.67 |
| HOMA-IR | 10.6±8.1 | 10.3±7.7 | 12.1±11.8 | 0.88 |
| Insulinogenic index | 29.1±19.4 | 30.1±20.2 | 38.1±22.8 | 0.069 |
| Disposition index | 3.6±2.8 | 3.7±2.8 | 4.8±3.6 | 0.19 |
| BIGTT-S_I_ | 9.2±4.1 | 9.4±4 | 9.2±4.4 | 0.29 |
| BIGTT-AIR | 1,838±1,070 | 1,879±1,176 | 2,381±1,042 | 0.066 |

Data are unadjusted mean±S.D data for a total of 5,787middle-aged individuals with either normal glucose tolerance (n = 4,381), impaired fasting glycemia (n = 491), impaired glucose tolerance (n = 667) or screen-detected and treatment-naïve type 2 diabetes (n = 248) stratified according to genotype. General linear regression analyses were used to calculate differences between geneotypes and p-values shown are for an additive genetic model and are adjusted for age, BMI and sex. incAUC, incremental area under the curve; HOMA-IR, homeostasis model assessment of insulin resistance; BIGTT-SI, BIGTT-insulin sensitivity; BIGTT-AIR, BIGTT acute insulin response.
