# Supplementary material for: The Type 2 Diabetes Associated Minor Allele of rs2237895 KCNQ1 Associates with Reduced Insulin Release Following an Oral Glucose Load
Source: PLoS One. 2009 Jun 11;4(6):e5872. doi: 10.1371/journal.pone.0005872 (PMC2689931; doi:10.1371/journal.pone.0005872)
Supplement: Table S7 — Anthropometrics and quantitative metabolic traits in the population-based Inter99 study sample in relation to the rs2237892 genotypes of KCNQ1. Data are unadjusted mean±S.D data for a total of 5,781 middle-aged individuals with either normal glucose tolerance (n = 4,381), impaired fasting glycemia (n = 483), impaired glucose tolerance (n = 672) or screen-detected and treatment-naïve type 2 diabetes (n = 245) stratified according to genotype. General linear regression analyses were used to calculate differences between geneotypes and p-values shown are for an additive genetic model and are adjusted for age, BMI and sex. incAUC, incremental area under the curve; HOMA-IR, homeostasis model assessment of insulin resistance; BIGTT-SI, BIGTT-insulin sensitivity; BIGTT-AIR, BIGTT acute insulin response. (0.03 MB DOC) [file pone.0005872.s007.doc]

Table S7: Anthropometrics and quantitative metabolic traits in the population-based Inter99 study sample in relation to the rs2237892 genotypes of *KCNQ1*.

| **rs2237892** | | | | |
| --- | --- | --- | --- | --- |
|  | CC | CT | TT | P _additive_ |
| N (m/w) | 5,079 (2,522/2,557) | 680 (340/340) | 22 (11/11) |  |
| Age (years) | 46±8 | 46±8 | 41±9 |  |
| BMI (kg/m^2^) | 26.2±4.6 | 26.1±4.5 | 25.1±4.7 | 0.45 |
| **Glucose traits** | | | | |
| Fasting p-glucose (mmol/l) | 5.5±0.8 | 5.5±0.8 | 5.1±0.7 | 0.036 |
| p-glucose at 30 min (mmol/l) | 8.7±1.9 | 8.7±1.9 | 8.1±1.5 | 0.90 |
| p-glucose at 120 min (mmol/l) | 6.2±2.1 | 6.1±2.1 | 5.7±2.0 | 0.12 |
| IncAUC glucose | 221±135 | 219±134 | 212±127 | 0.88 |
| **Insulin traits** | | | | |
| Fasting s-insulin (pmol/l) | 42±28 | 42±27 | 51±46 | 0.55 |
| s-insulin at 30 min (pmol/l) | 290±183 | 294±188 | 358±193 | 0.20 |
| s-insulin at 120 min (pmol/l) | 218±213 | 211±199 | 276±409 | 0.75 |
| IncAUC insulin | 22,904±15,991 | 22,725±15,121 | 29,387±22,974 | 0.47 |
| Fasting s-C-peptide (pmol/l) | 597±274 | 588±265 | 578±362 | 0.62 |
| C-peptide at 30 min (pmol/l) | 1,998±718 | 2,014±712 | 2,151±774 | 0.29 |
| C-peptide at 120 min (pmol/l) | 2,316±1,021 | 2,283±1,000 | 2,214±1,288 | 0.60 |
| IncAUC C-peptide (pmol/l) | 161,273±58,173 | 160,961±56,714 | 169,658±55,624 | 0.59 |
| HOMA-IR | 10.6±8.0 | 10.5±8.1 | 12.1±12.9 | 0.88 |
| Insulinogenic index | 29.2±19.4 | 30.1±20.5 | 37.0±19.6 | 0.28 |
| Disposition index | 3.6±2.8 | 3.7±2.7 | 5.0±3.9 | 0.35 |
| BIGTT-S_I_ | 9.2±4.1 | 9.4±4.0 | 9.6±4.5 | 0.39 |
| BIGTT-AIR | 1,839±1,072 | 1,886±1,155 | 2,362±865 | 0.12 |

Data are unadjusted mean±S.D data for a total of 5,781 middle-aged individuals with either normal glucose tolerance (n = 4,381), impaired fasting glycemia (n = 483), impaired glucose tolerance (n = 672) or screen-detected and treatment-naïve type 2 diabetes (n = 245) stratified according to genotype. General linear regression analyses were used to calculate differences between geneotypes and p-values shown are for an additive genetic model and are adjusted for age, BMI and sex. incAUC, incremental area under the curve; HOMA-IR, homeostasis model assessment of insulin resistance; BIGTT-SI, BIGTT-insulin sensitivity; BIGTT-AIR, BIGTT acute insulin response.
