# Supplementary material for: The Type 2 Diabetes Associated Minor Allele of rs2237895 KCNQ1 Associates with Reduced Insulin Release Following an Oral Glucose Load
Source: PLoS One. 2009 Jun 11;4(6):e5872. doi: 10.1371/journal.pone.0005872 (PMC2689931; doi:10.1371/journal.pone.0005872)
Supplement: Table S8 — D′ and r2 measures for the investigated KCNQ1 SNPs. Top triangle gives D′ and bottom triangle gives r2-values. (0.02 MB DOC) [file pone.0005872.s008.doc]

Table S8: D’ and r^2^ measures for the investigated *KCNQ1* SNPs

|  | rs2237892 | rs2283228 | rs2237895 | rs2237897 |
| --- | --- | --- | --- | --- |
| rs2237892 | - | 0.99 | 0.99 | 0.92 |
| rs2283228 | 0.80 | - | 0.76 | 0.93 |
| rs2237895 | 0.05 | 0.03 | - | 0.93 |
| rs2237897 | 0.57 | 0.48 | 0.03 | - |

Top triangle gives D’ and bottom triangle gives r^2^-values
